# The Mediating Effects of Food Content Watching Motivation on the between Watching Time and Nutrition Quotient of Adolescents in Seoul, Korea

**DOI:** 10.3390/nu14193901

**Published:** 2022-09-21

**Authors:** Da-Mee Kim, Bo-Mi Kim, Kyung-Hee Kim

**Affiliations:** Department of Food and Nutrition, Duksung Women’s University, 31 Samyang-ro, Dobong-gu, Seoul 01369, Korea

**Keywords:** food content, watching motivation, watching time, Nutrition Quotient for adolescents (NQ-A), adolescents, eating habits

## Abstract

Food-related content varies widely and is increasingly popular. Using various media, teenagers can easily access food content, which could affect they eating habits. This study was conducted to confirm the effects of watching motivation on the relationship between food content watching time and eating habits among adolescents in Seoul, Korea. Exactly 806 participants were surveyed about their food content watching status, including watching time and watching motivation. The Nutrition Quotient for adolescents (NQ-A) questionnaire was used to confirm eating habits. Exploratory factor analysis was conducted to classify watching motivation’s subfactors. A parallel multimedia model was used to analyze the effect of watching motivation on the relationship between food content watching time and eating habits. As a result of this study, following the factor analysis, watching motivation was classified into information acquisition, emotional satisfaction, and enjoyment. The influence of food content watching time on NQ-A scores through information acquisition motivation was positively significant, whereas that through emotional satisfaction motivation was negatively significant. Enjoyment motivation did not indirectly affect the relationship between food content watching time and NQ-A scores. Hence, attention should be paid to these mediating factors when analyzing the relationship between watching food-related content and eating habits. Developing and distributing content that meets viewing motivations should help improve adolescents’ eating habits.

## 1. Introduction

Adolescence is a period of rapid growth and development, which includes physical, psychosocial, sexual, and cognitive maturation [1]. Hence, it requires balanced nutritional intake [1,2]. The formation of a healthy diet is important at this stage because the nutritional status of adolescents can affect their lifelong health, including the onset of chronic diseases [3,4]. However, adolescents in many countries have an unhealthy diet, consuming more sugary drinks and junk food and fewer vegetables [5]. The formation of the eating habits of adolescents has a complex influence on individual influences, interpersonal relationships, families, community settings, and social environments (e.g., media and advertising) [6,7]. In particular, the media environment is among the major environmental factors that affect eating habits [8,9]. Adolescents’ eating or snacking while watching media influences the formation of undesirable eating habits, such as increased consumption of high-energy foods (e.g., salty food, fried food, soda, and sweets) and decreased consumption of fruits and vegetables [10,11,12,13,14]. Online food marketing aggressively targets adolescents and has been strongly associated with adolescent obesity and unhealthy eating habits [15,16,17]. Food content containing advertisements is recommended for voluntary regulation because it affects adolescents’ eating habits, but companies often do not comply with recommendations [18].

Food media content has recently become popular in Korea and worldwide [19]. Food-related content provides sensory satisfaction by maximizing the sound of eating food, giving people who are not eating a surrogate satisfaction, providing people who eat alone with a sense of togetherness, and giving social and emotional satisfaction [20]. Watching cooking videos gives viewers recipe ideas or motivates them to cook; it also sometimes leads to actual food purchases [21,22]. Access to food-related content through online channels has also increased [23,24]. As online content that shows overeating or unhealthy eating habits generates a higher number of views, it becomes more stimulating and increasingly popular [15,16,17,25]. Some teenagers search for food-related content for cooking inspiration, recipe information, and surrogate satisfaction, whereas some are exposed to food-related content by chance while using social networking services(SNS) [15,26].

The motivation to watch food-related content varies depending on the media platform. It may be to see partnerships, entertainment, etc. in traditional media; or to obtain information or inspiration, entice one’s appetite, experience social interaction, and appreciate aesthetic food in online media [26]. Watching motivation affects the selection of food-related content types, which can influence other aspects of youth eating habits [27,28]. Preventive nutrition education and nutritional intervention in adolescence have effects such as preventing obesity and improving nutritional status [29,30]. Identifying and improving the factors that affect adolescents’ eating habits can help improve their current and post-adult health.

Research on food-related content has been conducted, but most studies have only confirmed the relationship between the use of some content or social media and body image, eating disorders, food choices, and obesity [31,32,33,34,35]. More exploratory studies are needed because the relationship between the frequency of watching food-related content, watching motivation, and diet has been reported [25,27]. A recent study also reported that media watching affects adolescents’ dietary habits, and that wise use of media is necessary to form healthy eating habits [36]. However, the relationship between food-related content, among various contents types, and adolescents’ eating habits has not been studied yet. As the popularity and impact of food-related content are expected to continue for adolescents, the status of adolescents’ use of food content media should be investigated. In addition, it is necessary to understand the impact of adolescents’ food content watching time, eating habits, and watching motivation.

Therefore, the current study conducted parallel mediation analysis to verify the following hypotheses: (1) Among the watching motives, information acquisition is a significant mediating factor in the relationship between food content watching time and nutrition quotient. (2) Among the watching motives, emotional satisfaction is a significant mediating factor in the relationship between food content watching time and nutrition quotient. (3) Among the watching motives, enjoyment is a significant mediating factor in the relationship between food content watching time and nutrition quotient.

## 2. Materials and Methods

### 2.1. Participants and Procedure

A research notice was issued to middle and high schools in Seoul, and they received an application for participation from an institution capable of research cooperation. Four schools were selected by a simple random sampling method in which serial numbers were assigned to nine schools that applied for the study, and the samples were selected using a computer. Middle schools and high schools from both southern and northern Seoul were selected. A notice was posted at each participating school, and subjects were recruited by explaining the research purpose and investigation method. With the cooperation of the institutions’ nutrition teachers and homeroom teachers, the research manual and consent form were sent to each family, and consent forms from participants and guardians were collected through the institution. The subjects who met all criteria were included in the study. The inclusion criteria were: subjects aged 13–18 years who had watched food-related content in the past year. All participants and guardians listened to detailed explanations of the study and submitted consent forms before responding to the questionnaire. Research qualifications, including the participants’ ages and experience in watching food-related content, were reviewed through consent forms. Experience in watching food-related content and eating habits were investigated using a self-report questionnaire. The investigation period was from 1 May 2021, to 31 July 2021. Of the 1100 questionnaires, 1089 were retrieved. In the analysis, 806 responses were used while 172, 53, and 56 responses were excluded because they respectively showed that the respondents did not watch food-related content, failed to submit their signature and provide parental consent, and did not respond sincerely. All research procedures and methods were approved by the Institutional Review Board of Duksung Women’s University.

### 2.2. Measures

#### 2.2.1. Demographic Statistics

To analyze the basic data that could affect the use of food-related content and the eating habits of the participants, this study evaluated their gender, age, height, and weight as demographic sociological items. Body mass index (BMI) was calculated using height and weight.

#### 2.2.2. Experience in Watching Food-Related Content

In this study, food-related content was defined as including eating broadcast, cooking broadcasts, and food-themed content. Watching food-related content included direct access to the content or accidental encounters while watching TV or using SNS. The experience of watching food-related content over the past year was investigated. The investigation included, the type of content (mukbang: eating broadcast, cookbang: cooking broadcast, ASMR: autonomous sensory meridian response, SNS food image or video, others), the route of watching the content (YouTube, SNS, TV, internet broadcast, others), and the average watching time per day. The motivation for watching food-related content was evaluated using nine questions, which were revised and supplemented previous studies [27,37,38,39]. A 5-point Likert scale ranging from 1 = “never” to 5 = “always” was used to calculate the scores. Three factors were extracted through exploratory factor analysis, and the overall explanatory power was found to be 64.311% (Table 1). Factor 1 consisted of three items: “recipe information”, “new food ingredient information”, and “how to eat deliciously”. This factor was labeled as “information acquisition”; its explanatory power and Cronbach’s ɑ value were 23.227% and 0.771, respectively. Factor 2 consisted of four items: “vicarious satisfaction”, “food feels more delicious”, ”less lonely when eating alone”, and “stress relief”. This factor was labeled as “emotional satisfaction”; its explanatory power and Cronbach’s ɑ were 22.418% and 0.711, respectively. Factor 3 was composed of two items: “kill time” and “fun”. It was labeled as “enjoyment”; its explanatory power and Cronbach’s ɑ were 18.666% and 0.604, respectively.

#### 2.2.3. Nutrition Quotient for Adolescents (NQ-A)

The Nutrition Quotient for adolescents (NQ-A) is a checklist of the nutritional intake and dietary behavior of 13–18-year-olds developed by the Korean Nutrition Society [40]. It is used to comprehensively evaluate the quality and eating habits of individuals or groups. The NQ-A questionnaire is divided into five detailed factors: balance, diversity, moderation, environment, and practice. It comprises 19 items. The balance area refers to the frequency of intake of fruits, white milk, beans or bean products, and fish. The diversity area includes the number of vegetable side dishes, side dish diversity, and specific food rejection. The moderation area is the frequency of consumption of cookies, sweet and greasy bread, processed beverages, ramyeon, caffeine beverages, late-night snacks, and street foods. The environment area includes breakfast frequency, not moving around while eating, and screen time. The practice area includes checking nutrition labels, exercising frequency, and washing hands before meals. In this study, the NQ-A score was calculated using the scores and weights for each item following the method presented by Kim et al. (2017). The scores for each of the five areas were set to 100 points. The higher the total NQ-A score, the better the diet quality and eating habits.

### 2.3. Statistical Analysis

Statistical analysis was conducted using SPSS 26.0 (IBM, Chicago, IL, USA). Exploratory factor analysis was performed to verify the validity of the food content watching motivation variable used in this study. Factor analysis was performed using principal component analysis and varimax rotation. The number of factors with a reference eigenvalue of 1 or more was extracted, and the factor loading was found to be 0.4 or more. To analyze the reliability of the extracted factors, the Cronbach’s ɑ value, representing the internal consistency of the measurement items, was calculated.

Gender, food content watching type, and route were shown as frequency and percentage while age, BMI, average food content watching time, and NQ-A score were shown as mean and standard deviation. The food content watching time was converted using logs for normal distribution. Skewness and kurtosis were used to determine the distribution of data. The results confirmed that skewness (−0.61 to 1.10) and kurtosis (−0.57 to 1.96) were within the allowable range. Pearson correlation was performed to confirm the discriminant validity between the variables used in the study, and biserial correlation was performed for categorical variables, such as gender. Hayes’ parallel mediator model (model 4) was used in SPSS PROCESS MACRO version 4.1 to verify the mediating effect of food content viewing purpose (information acquisition, emotional satisfaction, and enjoyment) on the influence of food content watching time on the NQ-A scores. The independent variable was food content watching time, the dependent variable was the NQ-A score, and the mediation variable was food content watching motivation (i.e., information acquisition, emotional satisfaction, and enjoyment). The number of bootstrapping cases was set to 10,000. If 0 was not included in the 95% confidence interval of the indirect effect, it was determined that there was a mediating effect. BMI, gender, and age have been potentially associated with eating habits; therefore, they were used as covariates in this study.

## 3. Results

### 3.1. General Characteristics of Participants

The average age of the 806 subjects was 15.6 years (Table 2). Of the participants, 42.6% were male while 57.4% were female. Mukbang (eating broadcasts) was the most watched food-related content type at 50.0%, followed by cookbang (cooking broadcasts) at 30.1%, ASMR (autonomous sensory meridian response) at 11.7%, and social media food photos and videos at 7.2%. YouTube was the most popular route for watching content (80.8%). The average daily watching time was 47.3 min.

The Pearson correlation coefficients between food content viewing time, viewing motivation, NQ-A score, and covariates are presented in Table 3. If the correlation coefficient between variables was high (at 0.80 or higher), multicollinearity was considered. In this study, the correlation coefficient ranged from −0.346 to 0.450, and discriminant validity was confirmed.

### 3.2. Mediating Role of Food Content Watching Motives

**Figure 1:**
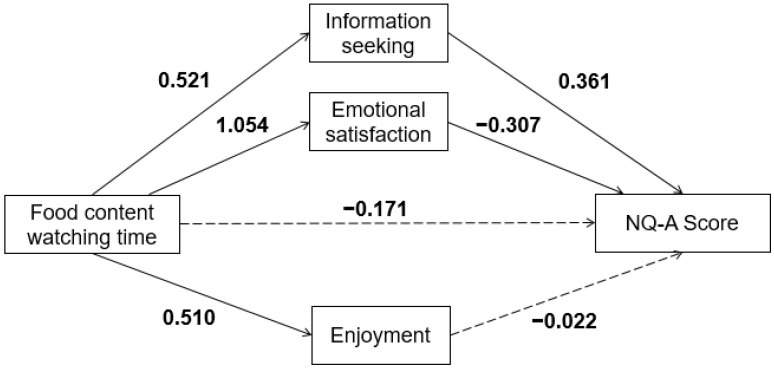
Mediation analysis with the hypothesized mediators of food content watching motives. Values indicate unstandardized coefficients from Hayes’ method. Gender, age and BMI are adjusted for the model. Food content watching timeis converted using logs for normal distribution. Values from food content watching time to Nutrition Quotient for adolescents (NQ-A) score are the direct effects of food content watching time to NQ-A score. Values from food content watching time to mediators (food content watching motives) are the direct effects offood content watching timeon mediators. Values from mediators to NQ-A score are the indirect effects of mediators on the relationship between food content watching motives and NQ-A score. →: Significant pathway, ⇢: Non-significant pathway.

Food content watching motivation (information acquisition, emotional satisfaction, and enjoyment) was a significant mediating factor in the relationship between food content watching time and NQ-A scores. By synthesizing these assumptions (Figure 1), a research model in which food content watching motivation mediates the relationship between food content watching time and NQ-A scores was developed.

Among the main paths of the mediating model, watching time → information acquisition (unstandardized coefficient [B] = 0.521, *p* < 0.001), watching time → emotional satisfaction (B = 1.054, *p* < 0.001), watching time → enjoyment (B = 0.510, *p* < 0.001), information acquisition → NQ-A score (B = 0.693, *p* < 0.001), and emotional satisfaction → NQ-A score (B = −0.291, *p* < 0.01) were significant, but the path of enjoyment → NQ-A score was not significant (Table 4).

Food content watching time did not directly affect the NQ-A scores (Table 5). Food content watching time did not change eating habit scores. However, among food content watching motives, information acquisition had a significant indirect effect on the relationship between watching time and NQ-A scores (B = 0.361, 95% CI = 0.174, 0.592). This means that the eating habit score increased as the watching time increased, due to the information acquisition viewing motivation acting as a mediating factor. Meanwhile, emotional satisfaction had a significant indirect effect on the relationship between watching time and NQ-A scores (B = −0.307, 95% CI = −0.608, −0.022). The emotional satisfaction mediating factor lowered the eating habit score as the food content watching time increased. The motivation for enjoyment did not act as a mediating factor between food content watching time and eating habis score. This was the result of the adjusted covariates, and it was significant even in the model that did not adjust for covariates. Among the mediating effects proposed in this study, the effects of information acquisition and emotional satisfaction on food content watching motivation supported the relationship between food content watching time and NQ-A scores.

## 4. Discussion

This study was conducted to confirm the effect of watching motivation on the relationship between food content watching time and eating habits among adolescents in Seoul. The motives for watching food-related content identified in this study were information acquisition, emotional satisfaction, and enjoyment. In previous studies on food content watching, the general watching motives were killing time, habitual viewing, entertainment, information acquisition, vicarious satisfaction, and personal relationship, all of which were related to the type of content [21,27,41].

The most watched types of content were mukbang and cookbang. Online mukbang content mainly consists of stimulating content, such as overeating, eating fast, eating spicy or stimulating food, and drinking together with food [20,25]. For adolescents, the online cooking broadcast platform provides recipe information or inspiration, which can affect cooking behavior [42]. Most of the adolescents’ food content watching routes were YouTube and social media, and online platforms were mainly used. Adolescents used online platforms more frequently than TVs [26,41]. The unrefined online media exposure of adolescents had a significant impact on emotions such as physical dissatisfaction [43]. Anyone can access online content that includes stimulating content, drinking, and advertising, and there are no age restrictions or special regulations [24,25,44,45]. The lower the grade level, the harder it may be to recognize that the advertisement notation is an advertisement [46]. Adolescents may be reluctant to view content directly designated as advertisements [47]. Considering the type and routes of content used by adolescents, legal restrictions should be imposed to limit stimulating content or food advertisements that have harmful effects.

The average daily watching time for food-related content was 47.3 min. Adolescents spend two to three hours a day using media such as the internet and social media [22,23]. This trend is increasing continuously [22]. We confirmed that watching food-related content accounts for a large portion of adolescents’ total media usage time. Other studies have reported that in adolescents, food media affected the formation of unhealthy eating habits and that excessive watching of food-related content may potentially promote eating disorders [10,11,48,49]. Food media strategically allows people to watch food-related content during weight loss to feel vicarious satisfaction, appetite suppression, and satiety [20,50]. In addition, food-related content is often used to find recipe information or inspiration [26]. The effect of watching food-related content on eating habits is not exactly one-sided; it could be helpful, harmful, or both [51,52]. The impact on eating habits may vary depending on the perspective adopted while consuming food-related content.

In this study, the relationship between food content watching time and NQ-A score was confirmed. The NQ-A score evaluates eating habits: the higher the score, the better the quality of the diet and eating habits [40]. There was no direct or total effect observed between food content watching time and NQ-A scores; thus, content watching time did not affect eating habits. This was different from the results of previous studies that showed that exposure and food content watching time exerted positive and negative effects on diet [10,52,53]. Nevertheless, it was possible to confirm the relationship between content watching time and eating habits as mediating factors for food content watching motivation. Watching food-related content as a motive for acquiring information, such as food recipe information, food ingredient information, and delicious eating methods, had a positive effect on increasing NQ-A scores as the watching time increased. In other words, when watching food content as a motive to acquire information, the eating habit score increased as the viewing time increased. Adolescents actively searched for food information, such as recipes and ingredients, prefering online platforms because they could easily obtain the desired information from them [26]. The motivation for information acquisition was primarily to select and watch cookbang content, which led to an increase in the desire for cooking; in fact, they often cooked and reported feeling that their eating habits improved [27,28].

Watching food-related content to satisfy emotional satisfaction, such as vicarious satisfaction, more delicious feelings, less loneliness when eating alone, and relieving stress, exerted a negative effect on lowering NQ-A scores and decreased the eating habit score as watching time increased. Depending on the watching motivation, the type of content and the impact on eating habits varied. In a study that identified watching motivation according to the type of food-related content, the motivation to watch mukbang was reported as vicarious satisfaction or killing time. [27,28,54]. Mukbang can satisfy emotional satisfaction motives; however, unrealistic visual and audio stimulation can reduce the pleasure received from actual meals [49]. The negative effects of media exposure on adolescents can be mitigated by critical thinking about harmful content [55]. It is necessary to limit the access of adolescents to provocative and harmful content. In addition, adolescents will need the ability to critically understand and accept the information provided by the content as well as the ability to access media.

Enjoyment as a motivation did not show a significant mediating effect on viewing time or NQ-A score. Watching food content with enjoyment as the motivation did not affect the eating habits score. Adolescents do not consume food-related content to satisfy their motivation for enjoyment if they accidentally watch food content while watching TV or using the internet [26]. This factor did not significantly affect the relationship between watching time and eating habits because food-related content was not actively consumed for enjoyment.

We believe that watching motivation plays an important role in content type selection rather than food content watching time, and that it affects eating habits. If watching motivation is satisfied after consuming a specific content, the preferred content can be continuously consumed [21]. Adolescents are growing up in an era where personal media use is common, and the effects of media viewing vary depending on the type, amount, scope, and individual characteristics of the media platform [56]. The proper use of food content viewing time or types can lead to improved eating habits or promote healthy behavior.

This study had several limitations. First, it was difficult to generalize the results because the study involved participants who were recruited only in parts of Seoul. In future studies, it will be necessary to consider whether this mediating model could be expanded by covering more regions and recruiting more participants. Second, it was difficult to confirm the causal relationships between the variables using a cross-sectional research design. For example, the mediating effect of increasing NQ-A scores as food content watching time increased through information acquisition motivation was confirmed, but the exact cause and the result could not be directly defined. The results of this study were insufficient for drawing conclusions on temporal sequences. Hence, additional research, designed over time is needed. Third, various confounding factors affecting eating habits were not collected or adjusted for. The interpretation of this study should be handled carefully considering the effects of unadjusted confounding factors. Fourth, most of the data collected in the study were self-reported, and there was a risk of bias, such as social desirability. It will be necessary to investigate the relationships identified in this study using more in-depth data collection tools and methods. Despite these limitations, to the best of our knowledge, this study was the first to investigate the mediating effects of watching motivation on the relationship between food content watching time and NQ-A scores. When adolescents cannot restrict their viewing of food-related content, attention should be paid to information acquisition and emotional satisfaction motives related to their eating habits. Rather than limiting the food content watching time of adolescents, content that satisfies their watching motivation should be produced. In addition, we believe that it is necessary to develop nutrition education programs that emphasize the importance of smart content consumption and healthy eating habits among adolescents.

## 5. Conclusions

This study confirmed that motivation for information acquisition, emotional satisfaction, and enjoyment exist when watching food-related content. In addition, an important mediating factor was identified between food content watching time and eating habit score. In the case of watching food-related content as a motive for obtaining food-related information, the eating habit score increased as watching time increased. When emotional satisfaction served as the mediating factor, the eating habit score decreased as the food content watching time increased. This meant that watching food-related content did not directly affect eating, but the effect of watching time on eating habits varied depending on the motivation for watching. Appropriate educational media and effective programs should be developed to correct adolescents’ eating habits. It will also be necessary to produce content focused on food ingredients, food, and food culture information to satisfy the motivation for information acquisition, as well as content focused on psychological stability and satisfaction to satisfy the motivation for emotional satisfaction. Such content will attract the interest of adolescents and increase the effectiveness of education, thereby contributing to the formation of proper eating habits.

## Figures and Tables

**Table 1:** Factor and reliability analysis of food-related content watching motives.

Type	Items	Factors		
		1	2	3
Information seeking	Food content provides me with recipe information	0.852		
	Food content provides me with new ingredient information	0.766		
	Food content provides me with information on how to eat deliciously	0.758		
Emotional satisfaction	While watching food content, I feel like I am eating, and I feel vicarious satisfaction		0.765	
	Watching food content while eating food makes the food seem more delicious		0.756	
	Watching food content while eating food makes you not feel lonely even if you eat alone		0.622	
	Watching food content relieves stress		0.584	
Enjoyment	I watch food content to kill time			0.808
	I watch food content for fun			0.801
	Eigenvalues	2.090	2.018	1.680
	% of variance	23.227	22.418	18.666
	Cumulative of %	23.227	45.646	64.311
	Cumulative of %	0.771	0.711	0.604
KMO = 0.801, Bartlett’s χ^2^ = 1946.443, df = 36, *p* < 0.001				

The Kaiser–Meyer–Olkin (KMO) method was used to measure sample appropriateness. Bartlett’s test of sphericity and total variance explained were used in the factor analysis evaluation.

**Table 2:** General characteristics of the subjects and the use of food-related content (N = 806).

	Mean ± SD or n(%)
Age (year)	15.6 ± 1.6
Gender	
Male	343 (42.6)
Female	463 (57.4)
BMI (kg/m^2^)	21.9 ± 4.0
Content type	
Mukbang	403 (50.0)
Cookbang	243 (30.1)
ASMR	94 (11.7)
SNS food image or video	57 (7.1)
Other	9 (1.1)
Route *	
YouTube	746 (80.8)
SNS	91 (9.9)
TV	59 (6.4)
Internet broadcast	24 (2.6)
Other	3 (0.3)
Food content watching time (min/day)	47.3 ± 51.1
NQ-A score	51.6 ± 10.8
Food content watching motives	
Information seeking	8.6 ± 2.9
Emotional satisfaction	11.2 ± 3.4
Enjoyment	6.9 ± 1.8

Values are presented as the mean ± standard deviation or number (percentage). Mukbang: online audiovisual broadcast in which a creator or host eats foods. ASMR (autonomous sensory meridian response): a video that stimulates hearing by maximizing the delicate sound of eating food. SNS: social network services. NQ-A: Nutrition Quotient for adolescents. * Multiple response available.

**Table 3:** Pearson’s correlation matrix of the variables of interest.

	1	2	3	4	5	6	7	8
1. Watching time	1	−0.022	0.181 ***	0.303 ***	0.282 ***	0.077 *	−0.042	0.073 *
2. NQ-A score		1	0.145 ***	−0.022	−0.001 ***	0.026	−0.051	−0.238 ***
3. Information seeking			1	0.450 ***	0.338 ***	0.088 *	−0.168 ***	0.019
4. Emotional satisfaction				1	0.399 ***	0.034	−0.125 ***	0.060
5. Enjoyment					1	0.022	−0.053	0.102 **
6. BMI						1	−0.346 ***	0.193 ***
7. Gender							1	−0.136 **
8. Age								1

Food content watching time was converted using logs for normal distribution. For categorical variables such as gender, the biserial correlation method was used. *** *p* < 0.001, ** *p* < 0.01, * *p* < 0.05

**Table 4:** Mediating effect pathway of food-related content watching motives in the relationship between food-related content watching time and nutritional quotient.

			B	S.E.	t	95%CI	
						LLCI	ULCI
IV to mediator							
Watching time	→	Information seeking	0.521	0.103	5.041 ***	0.318	0.723
Watching time	→	Emotional satisfaction	1.054	0.118	8.915 ***	0.822	1.286
Watching time	→	Enjoyment	0.510	0.062	8.204 ***	0.388	0.632
Mediator to DV							
Information seeking	→	NQ-A score	0.693	0.145	4.796 ***	0.409	0.977
Emotional satisfaction	→	NQ-A score	−0.291	0.128	−2.277 *	−0.542	−0.040
Enjoyment	→	NQ-A score	−0.043	0.232	−0.184	−0.499	0.414

Gender, age and BMI are adjusted for the model. Food content watching timeis converted using logs for normal distribution. IV: independent variable (Watching time), DV: dependent variable (NQ-A score), B: unstandardized coefficient. SE: standard error. LLCI: lower limit in 95% confidence interval. ULCI: upper in 95% confidence interval. *** *p* < 0.001, * *p* < 0.05

**Table 5:** Models of the effect of adolescents’ food content watching time on nutrition quotient with mediators of food content watching motives.

Variables							B	S.E.	95%CI	
									LLCI	ULCI
Total effect of IV on DV										
Watching time			→	NQ-A score			−0.138	0.383	−0.890	0.613
Direct effect of IV on DV										
Watching time			→	NQ-A score			−0.171	0.403	−0.962	0.621
Total indirect effect of IV on DV through proposed mediator							0.032	0.160	−0.281	0.349
Indirect effect of IV on DV through proposed mediator										
Watching time	→	Information seeking			→	NQ-A score	0.032	0.160	−0.281	0.349
Watching time	→	Emotional satisfaction			→	NQ-A score	0.032	0.160	−0.281	0.349
Watching time	→	Enjoyment			→	NQ-A score	−0.022	0.122	−0.266	0.218

Gender, age and BMI are adjusted for the model. Food content watching time is converted using logs for normal distribution. IV: independent variable (Watching time), DV: dependent variable (NQ-A score), B: unstandardized coefficient. SE: standard error. LLCI: lower limit in 95% confidence interval. ULCI: upper limit in 95% confidence interval. Bootstrap results for indirect effect.

## Data Availability

All data will be available upon requested.
